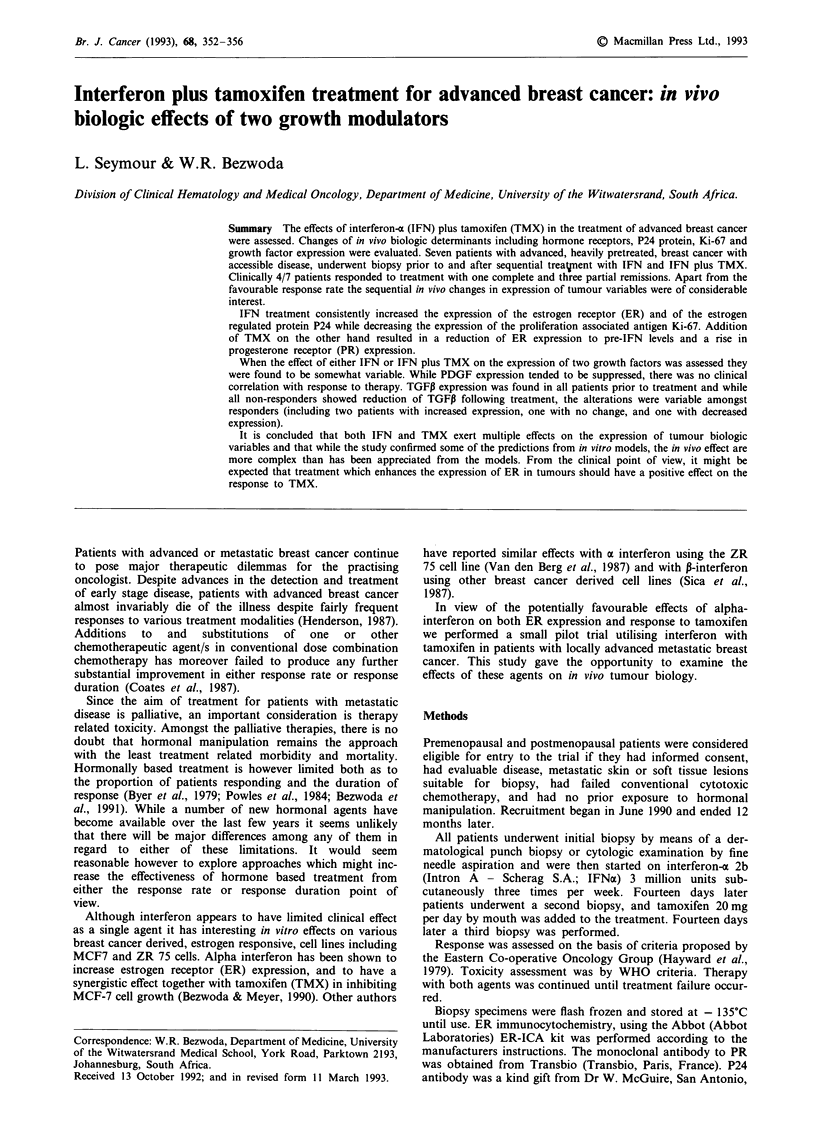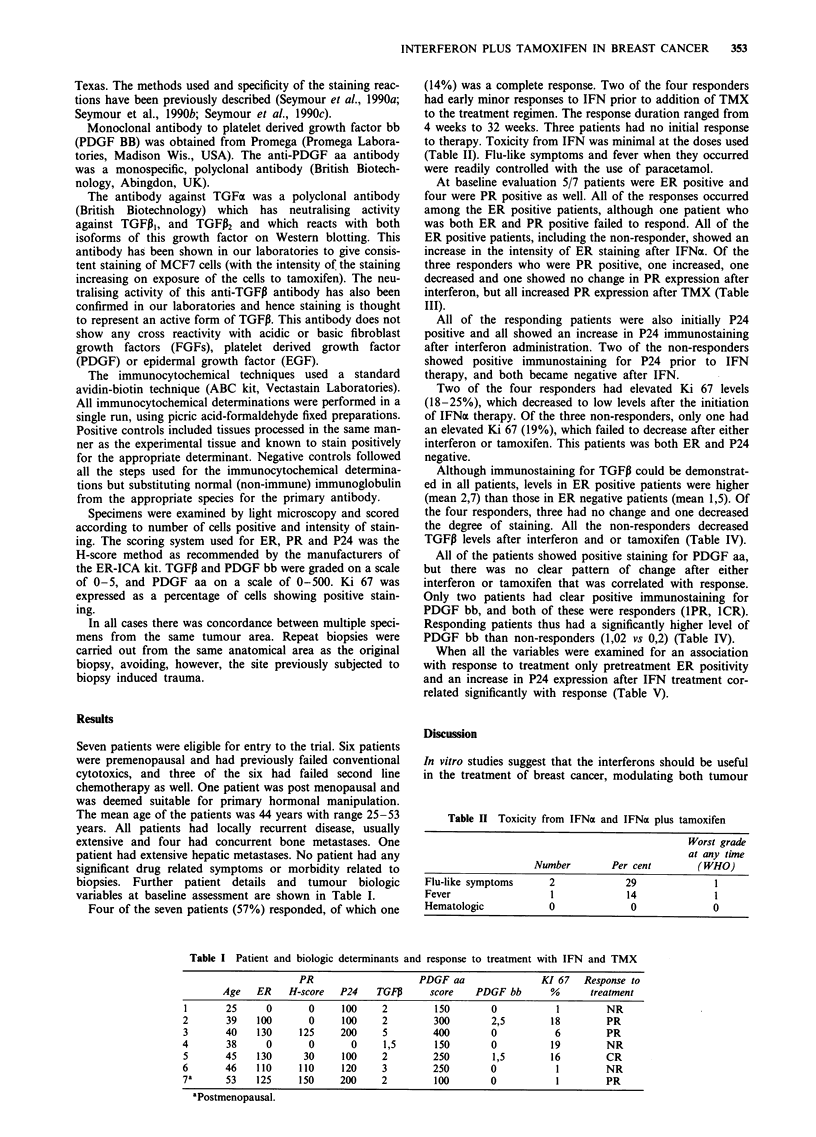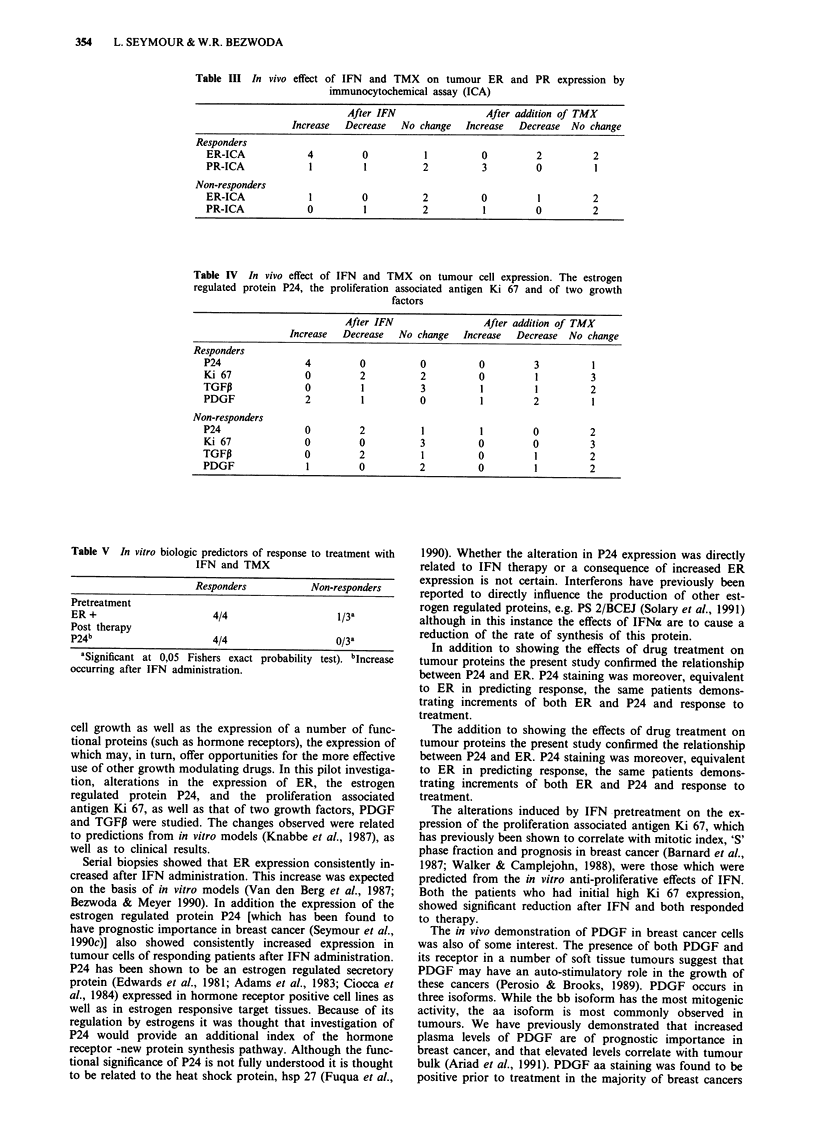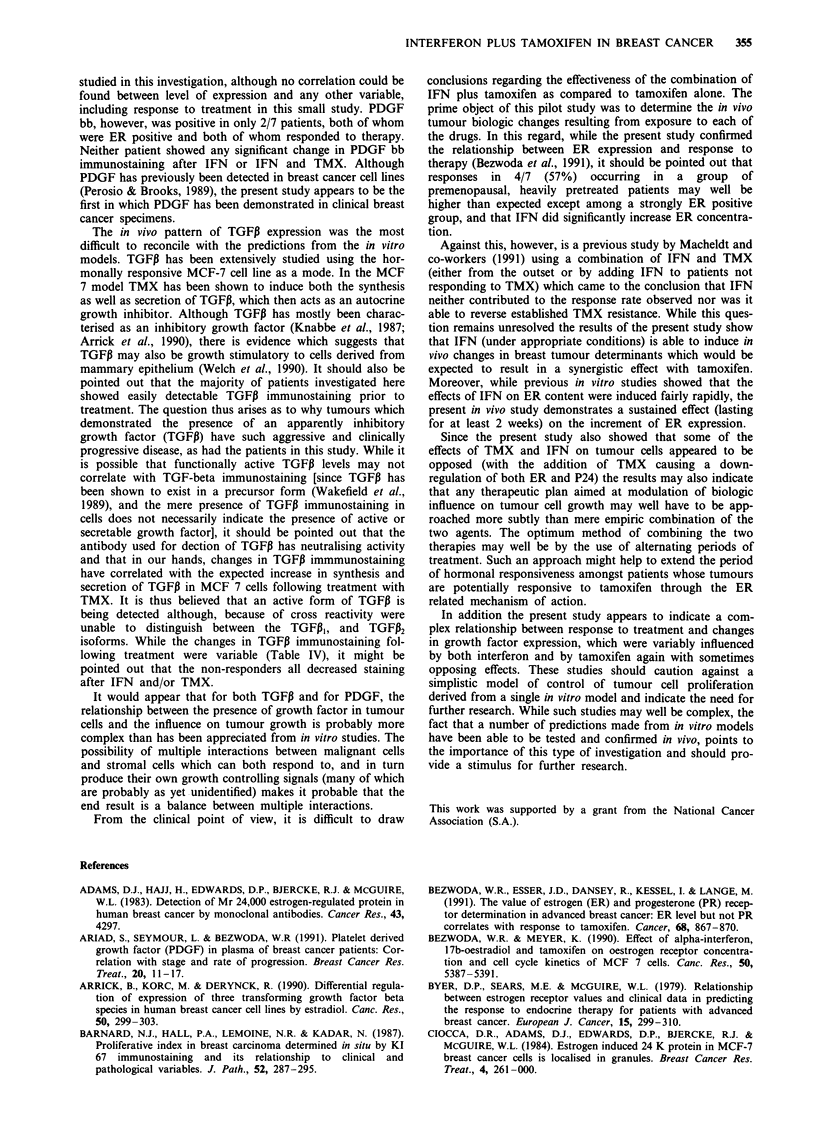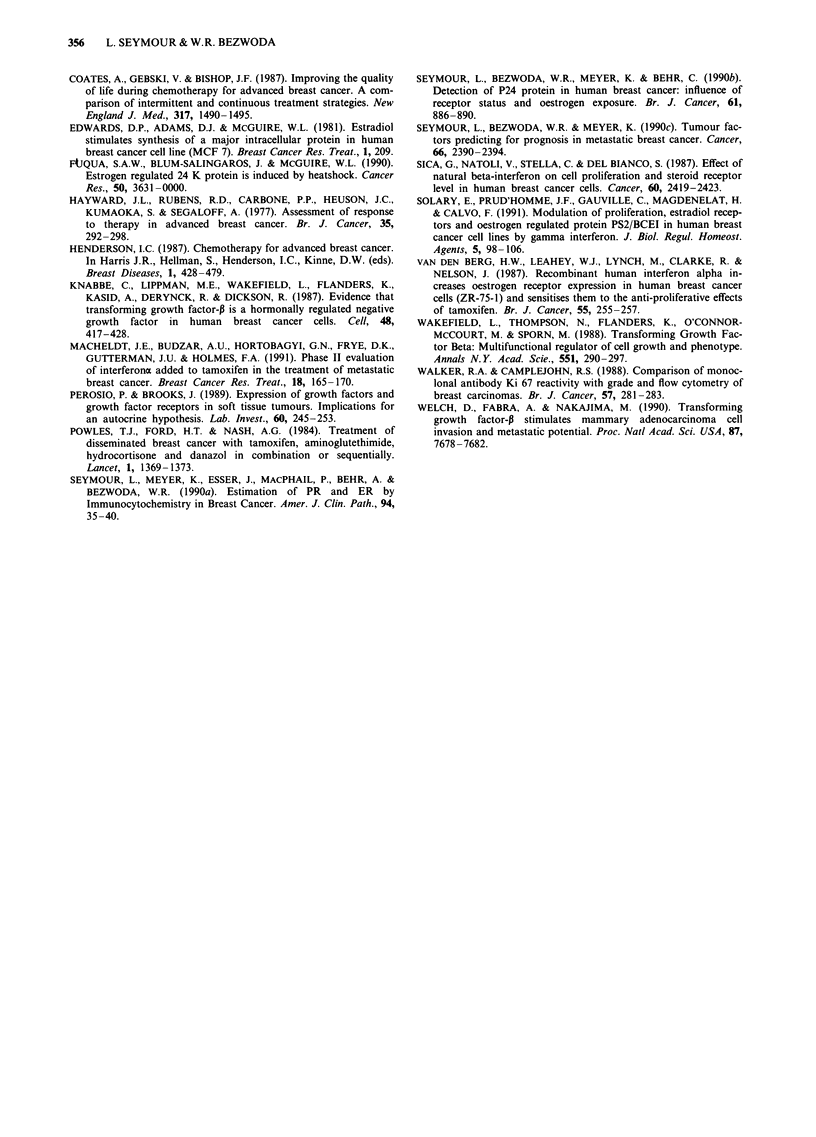# Interferon plus tamoxifen treatment for advanced breast cancer: in vivo biologic effects of two growth modulators.

**DOI:** 10.1038/bjc.1993.339

**Published:** 1993-08

**Authors:** L. Seymour, W. R. Bezwoda

**Affiliations:** Department of Medicine, University of the Witwatersrand, South Africa.

## Abstract

The effects of interferon-alpha (IFN) plus tamoxifen (TMX) in the treatment of advanced breast cancer were assessed. Changes of in vivo biologic determinants including hormone receptors, P24 protein, Ki-67 and growth factor expression were evaluated. Seven patients with advanced, heavily pretreated, breast cancer with accessible disease, underwent biopsy prior to and after sequential treatment with IFN and IFN plus TMX. Clinically 4/7 patients responded to treatment with one complete and three partial remissions. Apart from the favourable response rate the sequential in vivo changes in expression of tumour variables were of considerable interest. IFN treatment consistently increased the expression of the estrogen receptor (ER) and of the estrogen regulated protein P24 while decreasing the expression of the proliferation associated antigen Ki-67. Addition of TMX on the other hand resulted in a reduction of ER expression to pre-IFN levels and a rise in progesterone receptor (PR) expression. When the effect of either IFN or IFN plus TMX on the expression of two growth factors was assessed they were found to be somewhat variable. While PDGF expression tended to be suppressed, there was no clinical correlation with response to therapy. TGF beta expression was found in all patients prior to treatment and while all non-responders showed reduction of TGF beta following treatment, the alterations were variable amongst responders (including two patients with increased expression, one with no change, and one with decreased expression). It is concluded that both IFN and TMX exert multiple effects on the expression of tumour biologic variables and that while the study confirmed some of the predictions from in vitro models, the in vivo effect are more complex than has been appreciated from the models. From the clinical point of view, it might be expected that treatment which enhances the expression of ER in tumours should have a positive effect on the response to TMX.


*Br. J. Cancer* (1993), **68**, 352–356 © Macmillan Press Ltd., 1993

### Interferon plus tamoxifen treatment for advanced breast cancer: *in vivo* biologic effects of two growth modulators

L. Seymour & W.R. Bezwoda

*Division of Clinical Hematology and Medical Oncology, Department of Medicine, University of the Witwatersrand, South Africa.*

**Summary** The effects of interferon-α (IFN) plus tamoxifen (TMX) in the treatment of advanced breast cancer were assessed. Changes of *in vivo* biologic determinants including hormone receptors, P24 protein, Ki-67 and growth factor expression were evaluated. Seven patients with advanced, heavily pretreated, breast cancer with accessible disease, underwent biopsy prior to and after sequential treatment with IFN and IFN plus TMX. Clinically 4/7 patients responded to treatment with one complete and three partial remissions. Apart from the favourable response rate the sequential *in vivo* changes in expression of tumour variables were of considerable interest.

IFN treatment consistently increased the expression of the estrogen receptor (ER) and of the estrogen regulated protein P24 while decreasing the expression of the proliferation associated antigen Ki-67. Addition of TMX on the other hand resulted in a reduction of ER expression to pre-IFN levels and a rise in progesterone receptor (PR) expression.

When the effect of either IFN or IFN plus TMX on the expression of two growth factors was assessed they were found to be somewhat variable. While PDGF expression tended to be suppressed, there was no clinical correlation with response to therapy. TGFβ expression was found in all patients prior to treatment and while all non-responders showed reduction of TGFβ following treatment, the alterations were variable amongst responders (including two patients with increased expression, one with no change, and one with decreased expression).

It is concluded that both IFN and TMX exert multiple effects on the expression of tumour biologic variables and that while the study confirmed some of the predictions from *in vitro* models, the *in vivo* effect are more complex than has been appreciated from the models. From the clinical point of view, it might be expected that treatment which enhances the expression of ER in tumours should have a positive effect on the response to TMX.

Patients with advanced or metastatic breast cancer continue to pose major therapeutic dilemmas for the practising oncologist. Despite advances in the detection and treatment of early stage disease, patients with advanced breast cancer almost invariably die of the illness despite fairly frequent responses to various treatment modalities (Henderson, 1987). Additions to and substitutions of one or other chemotherapeutic agent/s in conventional dose combination chemotherapy has moreover failed to produce any further substantial improvement in either response rate or response duration (Coates *et al.*, 1987).

Since the aim of treatment for patients with metastatic disease is palliative, an important consideration is therapy related toxicity. Amongst the palliative therapies, there is no doubt that hormonal manipulation remains the approach with the least treatment related morbidity and mortality. Hormonally based treatment is however limited both as to the proportion of patients responding and the duration of response (Byer *et al.*, 1979; Powles *et al.*, 1984; Bezwoda *et al.*, 1991). While a number of new hormonal agents have become available over the last few years it seems unlikely that there will be major differences among any of them in regard to either of these limitations. It would seem reasonable however to explore approaches which might increase the effectiveness of hormone based treatment from either the response rate or response duration point of view.

Although interferon appears to have limited clinical effect as a single agent it has interesting *in vitro* effects on various breast cancer derived, estrogen responsive, cell lines including MCF7 and ZR 75 cells. Alpha interferon has been shown to increase estrogen receptor (ER) expression, and to have a synergistic effect together with tamoxifen (TMX) in inhibiting MCF-7 cell growth (Bezwoda & Meyer, 1990). Other authors have reported similar effects with α interferon using the ZR 75 cell line (Van den Berg *et al.*, 1987) and with β-interferon using other breast cancer derived cell lines (Sica *et al.*, 1987).

In view of the potentially favourable effects of alpha-interferon on both ER expression and response to tamoxifen we performed a small pilot trial utilising interferon with tamoxifen in patients with locally advanced metastatic breast cancer. This study gave the opportunity to examine the effects of these agents on *in vivo* tumour biology.

### Methods

Premenopausal and postmenopausal patients were considered eligible for entry to the trial if they had informed consent, had evaluable disease, metastatic skin or soft tissue lesions suitable for biopsy, had failed conventional cytotoxic chemotherapy, and had no prior exposure to hormonal manipulation. Recruitment began in June 1990 and ended 12 months later.

All patients underwent initial biopsy by means of a dermatological punch biopsy or cytologic examination by fine needle aspiration and were then started on interferon-α 2b (Intron A – Scherag S.A.; IFNα) 3 million units subcutaneously three times per week. Fourteen days later patients underwent a second biopsy, and tamoxifen 20 mg per day by mouth was added to the treatment. Fourteen days later a third biopsy was performed.

Response was assessed on the basis of criteria proposed by the Eastern Co-operative Oncology Group (Hayward *et al.*, 1979). Toxicity assessment was by WHO criteria. Therapy with both agents was continued until treatment failure occurred.

Biopsy specimens were flash frozen and stored at – 135°C until use. ER immunocytochemistry, using the Abbot (Abbot Laboratories) ER-ICA kit was performed according to the manufacturers instructions. The monoclonal antibody to PR was obtained from Transbio (Transbio, Paris, France). P24 antibody was a kind gift from Dr W. McGuire, San Antonio,

Correspondence: W.R. Bezwoda, Department of Medicine, University of the Witwatersrand Medical School, York Road, Parktown 2193, Johannesburg, South Africa.
Received 13 October 1992; and in revised form 11 March 1993.

Texas. The methods used and specificity of the staining reactions have been previously described (Seymour *et al.*, 1990*a*; Seymour et al., 1990*b*; Seymour *et al.*, 1990*c*).

Monoclonal antibody to platelet derived growth factor bb (PDGF BB) was obtained from Promega (Promega Laboratories, Madison Wis., USA). The anti-PDGF aa antibody was a monospecific, polyclonal antibody (British Biotechnology, Abingdon, UK).

The antibody against TGFα was a polyclonal antibody (British Biotechnology) which has neutralising activity against $TGF\beta_1$, and $TGF\beta_2$ and which reacts with both isoforms of this growth factor on Western blotting. This antibody has been shown in our laboratories to give consistent staining of MCF7 cells (with the intensity of the staining increasing on exposure of the cells to tamoxifen). The neutralising activity of this anti-TGFβ antibody has also been confirmed in our laboratories and hence staining is thought to represent an active form of TGFβ. This antibody does not show any cross reactivity with acidic or basic fibroblast growth factors (FGFs), platelet derived growth factor (PDGF) or epidermal growth factor (EGF).

The immunocytochemical techniques used a standard avidin-biotin technique (ABC kit, Vectastain Laboratories). All immunocytochemical determinations were performed in a single run, using picric acid-formaldehyde fixed preparations. Positive controls included tissues processed in the same manner as the experimental tissue and known to stain positively for the appropriate determinant. Negative controls followed all the steps used for the immunocytochemical determinations but substituting normal (non-immune) immunoglobulin from the appropriate species for the primary antibody.

Specimens were examined by light microscopy and scored according to number of cells positive and intensity of staining. The scoring system used for ER, PR and P24 was the H-score method as recommended by the manufacturers of the ER-ICA kit. TGFβ and PDGF bb were graded on a scale of 0–5, and PDGF aa on a scale of 0–500. Ki 67 was expressed as a percentage of cells showing positive staining.

In all cases there was concordance between multiple specimens from the same tumour area. Repeat biopsies were carried out from the same anatomical area as the original biopsy, avoiding, however, the site previously subjected to biopsy induced trauma.

### Results

Seven patients were eligible for entry to the trial. Six patients were premenopausal and had previously failed conventional cytotoxics, and three of the six had failed second line chemotherapy as well. One patient was post menopausal and was deemed suitable for primary hormonal manipulation. The mean age of the patients was 44 years with range 25–53 years. All patients had locally recurrent disease, usually extensive and four had concurrent bone metastases. One patient had extensive hepatic metastases. No patient had any significant drug related symptoms or morbidity related to biopsies. Further patient details and tumour biologic variables at baseline assessment are shown in Table I.

Four of the seven patients (57%) responded, of which one (14%) was a complete response. Two of the four responders had early minor responses to IFN prior to addition of TMX to the treatment regimen. The response duration ranged from 4 weeks to 32 weeks. Three patients had no initial response to therapy. Toxicity from IFN was minimal at the doses used (Table II). Flu-like symptoms and fever when they occurred were readily controlled with the use of paracetamol.

At baseline evaluation 5/7 patients were ER positive and four were PR positive as well. All of the responses occurred among the ER positive patients, although one patient who was both ER and PR positive failed to respond. All of the ER positive patients, including the non-responder, showed an increase in the intensity of ER staining after IFNα. Of the three responders who were PR positive, one increased, one decreased and one showed no change in PR expression after interferon, but all increased PR expression after TMX (Table III).

All of the responding patients were also initially P24 positive and all showed an increase in P24 immunostaining after interferon administration. Two of the non-responders showed positive immunostaining for P24 prior to IFN therapy, and both became negative after IFN.

Two of the four responders had elevated Ki 67 levels (18–25%), which decreased to low levels after the initiation of IFNα therapy. Of the three non-responders, only one had an elevated Ki 67 (19%), which failed to decrease after either interferon or tamoxifen. This patients was both ER and P24 negative.

Although immunostaining for TGFβ could be demonstrated in all patients, levels in ER positive patients were higher (mean 2,7) than those in ER negative patients (mean 1,5). Of the four responders, three had no change and one decreased the degree of staining. All the non-responders decreased TGFβ levels after interferon and or tamoxifen (Table IV).

All of the patients showed positive staining for PDGF aa, but there was no clear pattern of change after either interferon or tamoxifen that was correlated with response. Only two patients had clear positive immunostaining for PDGF bb, and both of these were responders (1PR, 1CR). Responding patients thus had a significantly higher level of PDGF bb than non-responders (1,02 *vs* 0,2) (Table IV).

When all the variables were examined for an association with response to treatment only pretreatment ER positivity and an increase in P24 expression after IFN treatment correlated significantly with response (Table V).

### Discussion

*In vitro* studies suggest that the interferons should be useful in the treatment of breast cancer, modulating both tumour

**Table II** Toxicity from IFNα and IFNα plus tamoxifen

| | *Number* | *Per cent* | *Worst grade at any time (WHO)* |
|---|---|---|---|
| Flu-like symptoms | 2 | 29 | 1 |
| Fever | 1 | 14 | 1 |
| Hematologic | 0 | 0 | 0 |

**Table I** Patient and biologic determinants and response to treatment with IFN and TMX

| | *Age* | *ER* | *PR H-score* | *P24* | *TGFβ* | *PDGF aa score* | *PDGF bb* | *KI 67 %* | *Response to treatment* |
|---|---|---|---|---|---|---|---|---|---|
| 1 | 25 | 0 | 0 | 100 | 2 | 150 | 0 | 1 | NR |
| 2 | 39 | 100 | 0 | 100 | 2 | 300 | 2,5 | 18 | PR |
| 3 | 40 | 130 | 125 | 200 | 5 | 400 | 0 | 6 | PR |
| 4 | 38 | 0 | 0 | 0 | 1,5 | 150 | 0 | 19 | NR |
| 5 | 45 | 130 | 30 | 100 | 2 | 250 | 1,5 | 16 | CR |
| 6 | 46 | 110 | 110 | 120 | 3 | 250 | 0 | 1 | NR |
| 7[a] | 53 | 125 | 150 | 200 | 2 | 100 | 0 | 1 | PR |

[a]Postmenopausal.

**Table III** *In vivo* effect of IFN and TMX on tumour ER and PR expression by immunocytochemical assay (ICA)

| | After IFN | | | After addition of TMX | | |
|---|---|---|---|---|---|---|
| | *Increase* | *Decrease* | *No change* | *Increase* | *Decrease* | *No change* |
| *Responders* | | | | | | |
| ER-ICA | 4 | 0 | 1 | 0 | 2 | 2 |
| PR-ICA | 1 | 1 | 2 | 3 | 0 | 1 |
| *Non-responders* | | | | | | |
| ER-ICA | 1 | 0 | 2 | 0 | 1 | 2 |
| PR-ICA | 0 | 1 | 2 | 1 | 0 | 2 |

**Table IV** *In vivo* effect of IFN and TMX on tumour cell expression. The estrogen regulated protein P24, the proliferation associated antigen Ki 67 and of two growth factors

| | After IFN | | | After addition of TMX | | |
|---|---|---|---|---|---|---|
| | *Increase* | *Decrease* | *No change* | *Increase* | *Decrease* | *No change* |
| *Responders* | | | | | | |
| P24 | 4 | 0 | 0 | 0 | 3 | 1 |
| Ki 67 | 0 | 2 | 2 | 0 | 1 | 3 |
| TGFβ | 0 | 1 | 3 | 1 | 1 | 2 |
| PDGF | 2 | 1 | 0 | 1 | 2 | 1 |
| *Non-responders* | | | | | | |
| P24 | 0 | 2 | 1 | 1 | 0 | 2 |
| Ki 67 | 0 | 0 | 3 | 0 | 0 | 3 |
| TGFβ | 0 | 2 | 1 | 0 | 1 | 2 |
| PDGF | 1 | 0 | 2 | 0 | 1 | 2 |

**Table V** *In vitro* biologic predictors of response to treatment with IFN and TMX

| | *Responders* | *Non-responders* |
|---|---|---|
| Pretreatment | | |
| ER + | 4/4 | 1/3[a] |
| Post therapy | | |
| P24[b] | 4/4 | 0/3[a] |

[a]Significant at 0,05 Fishers exact probability test). [b]Increase occurring after IFN administration.

cell growth as well as the expression of a number of functional proteins (such as hormone receptors), the expression of which may, in turn, offer opportunities for the more effective use of other growth modulating drugs. In this pilot investigation, alterations in the expression of ER, the estrogen regulated protein P24, and the proliferation associated antigen Ki 67, as well as that of two growth factors, PDGF and TGFβ were studied. The changes observed were related to predictions from *in vitro* models (Knabbe *et al.*, 1987), as well as to clinical results.

Serial biopsies showed that ER expression consistently increased after IFN administration. This increase was expected on the basis of *in vitro* models (Van den Berg *et al.*, 1987; Bezwoda & Meyer 1990). In addition the expression of the estrogen regulated protein P24 [which has been found to have prognostic importance in breast cancer (Seymour *et al.*, 1990*c*)] also showed consistently increased expression in tumour cells of responding patients after IFN administration. P24 has been shown to be an estrogen regulated secretory protein (Edwards *et al.*, 1981; Adams *et al.*, 1983; Ciocca *et al.*, 1984) expressed in hormone receptor positive cell lines as well as in estrogen responsive target tissues. Because of its regulation by estrogens it was thought that investigation of P24 would provide an additional index of the hormone receptor -new protein synthesis pathway. Although the functional significance of P24 is not fully understood it is thought to be related to the heat shock protein, hsp 27 (Fuqua *et al.*, 1990). Whether the alteration in P24 expression was directly related to IFN therapy or a consequence of increased ER expression is not certain. Interferons have previously been reported to directly influence the production of other estrogen regulated proteins, e.g. PS 2/BCEJ (Solary *et al.*, 1991) although in this instance the effects of IFNα are to cause a reduction of the rate of synthesis of this protein.

In addition to showing the effects of drug treatment on tumour proteins the present study confirmed the relationship between P24 and ER. P24 staining was moreover, equivalent to ER in predicting response, the same patients demonstrating increments of both ER and P24 and response to treatment.

The addition to showing the effects of drug treatment on tumour proteins the present study confirmed the relationship between P24 and ER. P24 staining was moreover, equivalent to ER in predicting response, the same patients demonstrating increments of both ER and P24 and response to treatment.

The alterations induced by IFN pretreatment on the expression of the proliferation associated antigen Ki 67, which has previously been shown to correlate with mitotic index, 'S' phase fraction and prognosis in breast cancer (Barnard *et al.*, 1987; Walker & Camplejohn, 1988), were those which were predicted from the *in vitro* anti-proliferative effects of IFN. Both the patients who had initial high Ki 67 expression, showed significant reduction after IFN and both responded to therapy.

The *in vivo* demonstration of PDGF in breast cancer cells was also of some interest. The presence of both PDGF and its receptor in a number of soft tissue tumours suggest that PDGF may have an auto-stimulatory role in the growth of these cancers (Perosio & Brooks, 1989). PDGF occurs in three isoforms. While the bb isoform has the most mitogenic activity, the aa isoform is most commonly observed in tumours. We have previously demonstrated that increased plasma levels of PDGF are of prognostic importance in breast cancer, and that elevated levels correlate with tumour bulk (Ariad *et al.*, 1991). PDGF aa staining was found to be positive prior to treatment in the majority of breast cancers studied in this investigation, although no correlation could be found between level of expression and any other variable, including response to treatment in this small study. PDGF bb, however, was positive in only 2/7 patients, both of whom were ER positive and both of whom responded to therapy. Neither patient showed any significant change in PDGF bb immunostaining after IFN or IFN and TMX. Although PDGF has previously been detected in breast cancer cell lines (Perosio & Brooks, 1989), the present study appears to be the first in which PDGF has been demonstrated in clinical breast cancer specimens.

The *in vivo* pattern of TGF$\beta$ expression was the most difficult to reconcile with the predictions from the *in vitro* models. TGF$\beta$ has been extensively studied using the hormonally responsive MCF-7 cell line as a mode. In the MCF 7 model TMX has been shown to induce both the synthesis as well as secretion of TGF$\beta$, which then acts as an autocrine growth inhibitor. Although TGF$\beta$ has mostly been characterised as an inhibitory growth factor (Knabbe *et al.*, 1987; Arrick *et al.*, 1990), there is evidence which suggests that TGF$\beta$ may also be growth stimulatory to cells derived from mammary epithelium (Welch *et al.*, 1990). It should also be pointed out that the majority of patients investigated here showed easily detectable TGF$\beta$ immunostaining prior to treatment. The question thus arises as to why tumours which demonstrated the presence of an apparently inhibitory growth factor (TGF$\beta$) have such aggressive and clinically progressive disease, as had the patients in this study. While it is possible that functionally active TGF$\beta$ levels may not correlate with TGF-beta immunostaining [since TGF$\beta$ has been shown to exist in a precursor form (Wakefield *et al.*, 1989), and the mere presence of TGF$\beta$ immunostaining in cells does not necessarily indicate the presence of active or secretable growth factor], it should be pointed out that the antibody used for deciton of TGF$\beta$ has neutralising activity and that in our hands, changes in TGF$\beta$ immmunostaining have correlated with the expected increase in synthesis and secretion of TGF$\beta$ in MCF 7 cells following treatment with TMX. It is thus believed that an active form of TGF$\beta$ is being detected although, because of cross reactivity were unable to distinguish between the TGF$\beta_1$, and TGF$\beta_2$ isoforms. While the changes in TGF$\beta$ immunostaining following treatment were variable (Table IV), it might be pointed out that the non-responders all decreased staining after IFN and/or TMX.

It would appear that for both TGF$\beta$ and for PDGF, the relationship between the presence of growth factor in tumour cells and the influence on tumour growth is probably more complex than has been appreciated from *in vitro* studies. The possibility of multiple interactions between malignant cells and stromal cells which can both respond to, and in turn produce their own growth controlling signals (many of which are probably as yet unidentified) makes it probable that the end result is a balance between multiple interactions.

From the clinical point of view, it is difficult to draw conclusions regarding the effectiveness of the combination of IFN plus tamoxifen as compared to tamoxifen alone. The prime object of this pilot study was to determine the *in vivo* tumour biologic changes resulting from exposure to each of the drugs. In this regard, while the present study confirmed the relationship between ER expression and response to therapy (Bezwoda *et al.*, 1991), it should be pointed out that responses in 4/7 (57%) occurring in a group of premenopausal, heavily pretreated patients may well be higher than expected except among a strongly ER positive group, and that IFN did significantly increase ER concentration.

Against this, however, is a previous study by Macheldt and co-workers (1991) using a combination of IFN and TMX (either from the outset or by adding IFN to patients not responding to TMX) which came to the conclusion that IFN neither contributed to the response rate observed nor was it able to reverse established TMX resistance. While this question remains unresolved the results of the present study show that IFN (under appropriate conditions) is able to induce *in vivo* changes in breast tumour determinants which would be expected to result in a synergistic effect with tamoxifen. Moreover, while previous *in vitro* studies showed that the effects of IFN on ER content were induced fairly rapidly, the present *in vivo* study demonstrates a sustained effect (lasting for at least 2 weeks) on the increment of ER expression.

Since the present study also showed that some of the effects of TMX and IFN on tumour cells appeared to be opposed (with the addition of TMX causing a down-regulation of both ER and P24) the results may also indicate that any therapeutic plan aimed at modulation of biologic influence on tumour cell growth may well have to be approached more subtly than mere empiric combination of the two agents. The optimum method of combining the two therapies may well be by the use of alternating periods of treatment. Such an approach might help to extend the period of hormonal responsiveness amongst patients whose tumours are potentially responsive to tamoxifen through the ER related mechanism of action.

In addition the present study appears to indicate a complex relationship between response to treatment and changes in growth factor expression, which were variably influenced by both interferon and by tamoxifen again with sometimes opposing effects. These studies should caution against a simplistic model of control of tumour cell proliferation derived from a single *in vitro* model and indicate the need for further research. While such studies may well be complex, the fact that a number of predictions made from *in vitro* models have been able to be tested and confirmed *in vivo*, points to the importance of this type of investigation and should provide a stimulus for further research.

This work was supported by a grant from the National Cancer Association (S.A.).

### References

ADAMS, D.J., HAJJ, H., EDWARDS, D.P., BJERCKE, R.J. & MCGUIRE, W.L. (1983). Detection of Mr 24,000 estrogen-regulated protein in human breast cancer by monoclonal antibodies. *Cancer Res.*, **43**, 4297.

ARIAD, S., SEYMOUR, L. & BEZWODA, W.R (1991). Platelet derived growth factor (PDGF) in plasma of breast cancer patients: Correlation with stage and rate of progression. *Breast Cancer Res. Treat.*, **20**, 11-17.

ARRICK, B., KORC, M. & DERYNCK, R. (1990). Differential regulation of expression of three transforming growth factor beta species in human breast cancer cell lines by estradiol. *Canc. Res.*, **50**, 299-303.

BARNARD, N.J., HALL, P.A., LEMOINE, N.R. & KADAR, N. (1987). Proliferative index in breast carcinoma determined *in situ* by KI 67 immunostaining and its relationship to clinical and pathological variables. *J. Path.*, **52**, 287-295.

BEZWODA, W.R., ESSER, J.D., DANSEY, R., KESSEL, I. & LANGE, M. (1991). The value of estrogen (ER) and progesterone (PR) receptor determination in advanced breast cancer: ER level but not PR correlates with response to tamoxifen. *Cancer*, **68**, 867-870.

BEZWODA, W.R. & MEYER, K. (1990). Effect of alpha-interferon, 17b-oestradiol and tamoxifen on oestrogen receptor concentration and cell cycle kinetics of MCF 7 cells. *Canc. Res.*, **50**, 5387-5391.

BYER, D.P., SEARS, M.E. & MCGUIRE, W.L. (1979). Relationship between estrogen receptor values and clinical data in predicting the response to endocrine therapy for patients with advanced breast cancer. *European J. Cancer*, **15**, 299-310.

CIOCCA, D.R., ADAMS, D.J., EDWARDS, D.P., BJERCKE, R.J. & MCGUIRE, W.L. (1984). Estrogen induced 24 K protein in MCF-7 breast cancer cells is localised in granules. *Breast Cancer Res. Treat.*, **4**, 261-000.

COATES, A., GEBSKI, V. & BISHOP, J.F. (1987). Improving the quality of life during chemotherapy for advanced breast cancer. A comparison of intermittent and continuous treatment strategies. *New England J. Med.*, **317**, 1490–1495.

EDWARDS, D.P., ADAMS, D.J. & MCGUIRE, W.L. (1981). Estradiol stimulates synthesis of a major intracellular protein in human breast cancer cell line (MCF 7). *Breast Cancer Res. Treat.*, **1**, 209.

FUQUA, S.A.W., BLUM-SALINGAROS, J. & MCGUIRE, W.L. (1990). Estrogen regulated 24 K protein is induced by heatshock. *Cancer Res.*, **50**, 3631–0000.

HAYWARD, J.L., RUBENS, R.D., CARBONE, P.P., HEUSON, J.C., KUMAOKA, S. & SEGALOFF, A. (1977). Assessment of response to therapy in advanced breast cancer. *Br. J. Cancer*, **35**, 292–298.

HENDERSON, I.C. (1987). Chemotherapy for advanced breast cancer. In Harris J.R., Hellman, S., Henderson, I.C., Kinne, D.W. (eds). *Breast Diseases*, **1**, 428–479.

KNABBE, C., LIPPMAN, M.E., WAKEFIELD, L., FLANDERS, K., KASID, A., DERYNCK, R. & DICKSON, R. (1987). Evidence that transforming growth factor-$\beta$ is a hormonally regulated negative growth factor in human breast cancer cells. *Cell*, **48**, 417–428.

MACHELDT, J.E., BUDZAR, A.U., HORTOBAGYI, G.N., FRYE, D.K., GUTTERMAN, J.U. & HOLMES, F.A. (1991). Phase II evaluation of interferon$\alpha$ added to tamoxifen in the treatment of metastatic breast cancer. *Breast Cancer Res. Treat.*, **18**, 165–170.

PEROSIO, P. & BROOKS, J. (1989). Expression of growth factors and growth factor receptors in soft tissue tumours. Implications for an autocrine hypothesis. *Lab. Invest.*, **60**, 245–253.

POWLES, T.J., FORD, H.T. & NASH, A.G. (1984). Treatment of disseminated breast cancer with tamoxifen, aminoglutethimide, hydrocortisone and danazol in combination or sequentially. *Lancet*, **1**, 1369–1373.

SEYMOUR, L., MEYER, K., ESSER, J., MACPHAIL, P., BEHR, A. & BEZWODA, W.R. (1990*a*). Estimation of PR and ER by Immunocytochemistry in Breast Cancer. *Amer. J. Clin. Path.*, **94**, 35–40.

SEYMOUR, L., BEZWODA, W.R., MEYER, K. & BEHR, C. (1990*b*). Detection of P24 protein in human breast cancer: influence of receptor status and oestrogen exposure. *Br. J. Cancer*, **61**, 886–890.

SEYMOUR, L., BEZWODA, W.R. & MEYER, K. (1990*c*). Tumour factors predicting for prognosis in metastatic breast cancer. *Cancer*, **66**, 2390–2394.

SICA, G., NATOLI, V., STELLA, C. & DEL BIANCO, S. (1987). Effect of natural beta-interferon on cell proliferation and steroid receptor level in human breast cancer cells. *Cancer*, **60**, 2419–2423.

SOLARY, E., PRUD'HOMME, J.F., GAUVILLE, C., MAGDENELAT, H. & CALVO, F. (1991). Modulation of proliferation, estradiol receptors and oestrogen regulated protein PS2/BCEI in human breast cancer cell lines by gamma interferon. *J. Biol. Regul. Homeost. Agents*, **5**, 98–106.

VAN DEN BERG, H.W., LEAHEY, W.J., LYNCH, M., CLARKE, R. & NELSON, J. (1987). Recombinant human interferon alpha increases oestrogen receptor expression in human breast cancer cells (ZR-75-1) and sensitises them to the anti-proliferative effects of tamoxifen. *Br. J. Cancer*, **55**, 255–257.

WAKEFIELD, L., THOMPSON, N., FLANDERS, K., O'CONNOR-MCCOURT, M. & SPORN, M. (1988). Transforming Growth Factor Beta: Multifunctional regulator of cell growth and phenotype. *Annals N.Y. Acad. Scie.*, **551**, 290–297.

WALKER, R.A. & CAMPLEJOHN, R.S. (1988). Comparison of monoclonal antibody Ki 67 reactivity with grade and flow cytometry of breast carcinomas. *Br. J. Cancer*, **57**, 281–283.

WELCH, D., FABRA, A. & NAKAJIMA, M. (1990). Transforming growth factor-$\beta$ stimulates mammary adenocarcinoma cell invasion and metastatic potential. *Proc. Natl Acad. Sci. USA*, **87**, 7678–7682.